# Pulmonary effects of ozone therapy at different doses combined with antibioticotherapy in experimental sepsis model^1^

**DOI:** 10.1590/s0102-865020200060000004

**Published:** 2020-07-13

**Authors:** Hasan Oğuz Kapicibaşi, Hasan Ali Kiraz, Emin Tunç Demir, Yasemen Adali, Sait Elmas

**Affiliations:** I Assistant Professor, Çanakkale Onsekiz Mart University, Faculty of Medicine, Department of Thoracic Surgery, Çanakkale, Turkey. Design of the study, technical procedures, manuscript writing, critical revision, final approval.; II Assistant Professor, Çanakkale Onsekiz Mart University, Faculty of Medicine, Department of Anesthesiology and Reanimation, Çanakkale, Turkey. Design of the study, technical procedures, manuscript writing, critical revision, final approval.; III MD, Trakya University, Faculty of Medicine, Department of Anesthesiology and Reanimation, Division of Intensive Care, Edirne, Turkey. Technical procedures, animal care, critical revision, final approval.; IV Associate Professor, Çanakkale Onsekiz Mart University, Faculty of Medicine, Department of Medical Pathology, Çanakkale, Turkey. Analysis and interpretation of data, histopathological examinations, critical revision, final approval.; V Veterinarian Doctor, Çanakkale Onsekiz Mart University, Experimental Research Application and Research Center, Çanakkale, Turkey. Technical procedures, animal care, critical revision, final approval.

**Keywords:** Lung, Ozone, Sepsis, Rats

## Abstract

**Purpose:**

This experimental sepsis model created with *Escherichia coli* aimed to investigate the histopathological effects of two different doses of ozone combined with antibiotherapy on lung tissue.

**Methods:**

Rats were divided into 5 groups. Then sepsis was induced intraperitoneally in the first 4 groups. The 1^st^ group was treated with cefepime, the 2^nd^ and 3^rd^ groups were treated with cefepime combined with ozone at a dose of 0.6 mg/kg and 1.1 mg/kg. Lung tissue sections were stained with hematoxylin-eosin and assessed under light microscope and scored between 0-4 in terms of histopathological findings.

**Results:**

In the comparisons between Group 1 and Group 4 in terms of cellular damage (p=0.030), inflammation (p=0.000) and overall score (p=0.007), statistically significant positive effects were observed in favor of Group 1. In the comparisons of Groups 2 and 3 with Group 4, only positive effects were observed in terms of inflammation (p=0.020, p=0.012, respectively).

**Conclusion:**

Although negative histopathological effects of ozone on tissue injury were detected, it was noteworthy that the increase in the ozone dose reduced the number of damaged parameters.

## Introduction

Sepsis is defined as life-threatening organ dysfunction caused by an unregulated host response to infection^1^. The pathology of sepsis involves complex interactions between host organs and invading pathogens. Ultimately tissue injury and organ failure are due to negative effects of systemic activation of host immunity^2,3^. Sepsis forms a large problem in intensive care and is the main cause of death occurring there^4,5^. According to a report published by the Global Burden of Disease, each year nearly 10 million people die from infections and this is much greater than the number of people who die from cancer annually^6^. Lungs are the first organs affected by sepsis and sepsis causes severe injury to lung tissue^7^. In spite of advanced antibiotherapy, supportive treatments and all technological opportunities, sepsis continues to be a situation progressing with morbidity and mortality^8^. The earliest target-directed basic treatment principles for sepsis comprise determining high-risk patients, ensuring appropriate cultures and source control, and beginning appropriate antibiotherapy without delay^9^.

Medical ozone therapy is an alternative treatment model linked to the administration of ozone and gas mixture with oxygen to body fluids or cavities. To date, clinical and experimental studies have shown that ozone therapy is beneficial for inflammation-mediated diseases like infected wounds, chronic skin ulcers, burns and advanced ischemic diseases^10^. Ozone/oxygen mixtures are reported to display a variety of effects on the immune system like phagocytic activity modulation^11^. Additionally, ozone therapy is proposed to cause increased antioxidant enzyme expression^12^. Additionally, it is known that ozone is also an immune system modulator. Though antiseptic properties of ozone are well known, it may harm not just the pathogen but also the patient linked to dose and exposure duration^13^. Ozone is one of the strongest oxidants and may show unwanted effects through a variety of mechanisms like free radical formation, lipid peroxidation, enzymatic activity loss, changes in membrane permeability, direct organ inflammation and injury^14^. Based on the known positive and negative effects of ozone, in our study we aimed to assess the effect of two different doses of ozone therapy added to antibiotic treatment in an experimental sepsis model induced with *Escherichia coli* on the histopathologic findings observed in the inflammatory process in the lungs.

## Methods

Ethical approval was obtained from Çanakkale Onsekiz Mart University Ethical Board of Animal Studies (File Registration Number: 2018/1800080971 & Decision number: 2018/06-06). Our study included 40 male Sprague-Dawley rats with mean weight 350-400 g in appropriate condition. During the study, rats were kept in ÇOMÜ Experimental Research Center in wire cages with 12-hour night-12-hour day circadian rhythm, environmental temperature 24-26°C and humidity 50-60%. Rats were fed with standard commercial feed and municipal drinking water. Rat feed was stopped 12 hours before the study; however, water was given freely during this time. All rat care was performed in accordance with the “Regulation on the Welfare and Protection of Animals Used for Experimental and Other Scientific Purposes” (13.12.2011-28141) prepared by the Ministry of Food, Agriculture and Livestock.

### *Experimental groups*

Experimental animals were randomly divided into 5 groups with equal numbers (n=8). Sepsis was induced in rats in the first 4 of these groups with 2.1x10^9^ CFU/mL *Escherichia coli* ATCC 25922 administered in 1 mL saline via the intraperitoneal route in 32 rats. Rats in the 5^th^ group only had 1 mL intraperitoneal saline administered.

### *Implementation and surgical procedure*

Twenty-four hours after *Escherichia coli* administration, rats in the 1^st^, 2^nd^ and 3^rd^ groups were arranged in groups following skin cleaning for asepsis and the following treatments began. Care was taken to administer treatments at the same time every day. Additionally, an ozone generator (device name and model: Turkozone Blue S, device serial no: BG-19144427046) was used to produce the ozone/oxygen mixture for ozone therapy.

**1** ^st^ **group:** This group was the antibiotic group, so after *Escherichia coli* was administered by the intraperitoneal route with the aim of inducing sepsis, cefepime 50 mg/kg dose was administered subcutaneously at the same time for 7 days.

**2** ^nd^ **group:** Rats in this group had single dose of 0.6 mg/kg ozone therapy administered for 1 min intraperitoneal simultaneous to the 50 mg/kg subcutaneous cefepime for 7 days.

**3** ^rd^ **group:** Rats in this group had single dose of 1.1 mg/kg ozone therapy, different to the 2^nd^ group, administered for 1 min intraperitoneal simultaneous to the 50 mg/kg subcutaneous cefepime for 7 days.

**4** ^th^ **group:** Rats in this group were not given any treatment after administration of *Escherichia coli* by the intraperitoneal route with the aim of inducing sepsis.

**5** ^th^ **group:** Rats in this group did not have a sepsis model induced, but were administered 1 mL saline intraperitoneal at the start and were only administered subcutaneous saline at equivalent times to the treatments in the other groups.

**Figure 1:**
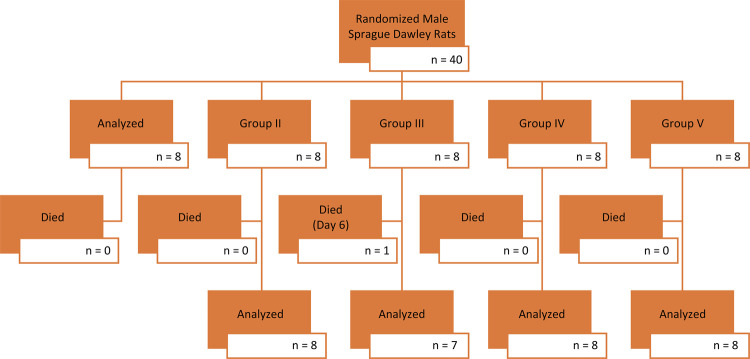
Study flow chart.

Twenty-four hours after the final treatment in our study, rats were administered high-dose anesthetics (xylazine - 10 mg/kg and ketamine - 80 mg/kg) and lung tissue samples and blood samples were taken by accessing the thorax cavity with median sternotomy and rats were sacrificed. In the blood samples, the diagnosis of sepsis was confirmed by working with the rats *Escherichia coli* protein (*E.coli P*) enzyme-linked immunosorbent assays (ELISA) kits (Shangai Coon Koon Biotech Co, China). In view of the data on the researched serums, the very low level of *E.coli P* in the rats of the control group was construed and reported as the absence of sepsis. The statistically significant differences concerning the presence of *E.coli P* in the first four groups in comparison with the control groups signify that sepsis was successfully induced in these groups (Fig. 1).

Lung tissue samples were fixated for 24 hours in 10% buffered formalin solution. Later, tissue monitoring procedures were performed appropriate to groups and tissues were submerged in paraffin. Sections with 4-micron thickness were taken from the prepared paraffin blocks and stained with hematoxylin & eosin (H&E) before being investigated with a light microscope (Olympus BX46, Japan) at magnification above 10 fields^15^.

The tissues were assessed by the parameters used by Yamanel *et al*.^16^ with some modification and their scoring system was used. Hemorrhage, cellular injury, alveoloseptal thickening and inflammation were scored from 0-4 and a general injury score (total score) was obtained by summing all scores per case.

### *Statistical analysis*

Maximum care was taken to use the lowest number of animals necessary to gain scientific quality from the methods applied in the study and the obtained results. Analysis of data used the Statistical Package for the Social Sciences for Windows Evaluation v. 15.0 (SPSS, Chicago, IL, USA) program. Results are given as median and minimum-maximum values. Comparisons of groups used the Mann-Whitney U test. Tests were completed in the 95% confidence interval and values with p lower than 0.05 were accepted as statistically significant.

### *Histopathological findings*

As the histopathologic findings observed in the study are categoric data, the median and interval values are presented in Table 1. When Group 4 is compared with the control group of Group 5, Group 4 was identified to have more statistically significant injury in terms of cellular damage (p=0.030), alveoloseptal thickening (p=0.037), inflammation (p=0.001) and total score (p=0.009), with no significant difference between the groups in terms of hemorrhage (p=0.663).

**Table 1:** Statistical significance with value intervals and median values for histopathological findings.

	Group 1 Median (Min-Max)	Group 2 Median (Min-Max)	Group 3* Median (Min-Max)	Group 4 Median (Min-Max)	Group 5 Median (Min-Max)
Bleeding	2/2 (1-3)	1/1 (1-4)	2 (1-3)	2/2 (1-4)	2/2 (1-4)
Cellular Damage	0/0 (0-1)^b^	1/1 (1-3)^a,c^	2 (1-3)^a,c^	1/1 (0-3)^a,c^	0/0 (0-1)^b^
Alveoloseptal Thickening	0/0 (0-1)	1/2 (0-3)^a,c^	1 (0-3)^a^	1/1 (0-3)^a^	0/0 (0-1)^b^
Inflammation	0/0 (0-1)^b^	1/1 (1-3)^a,b,c^	1 (0-3)^b^	2/3 (2-4)^a,c^	0/0 (0-1)^b^
Total Score	2/2 (1-5)^b^	5/8 (3-11)^a,c^	6 (2-11)^c^	7/8 (3-12)^a,c^	3/3 (1-7)^b^

^a^: p<0.05, when compared with Group 5, ^b^: p<0.05, when compared with Group 4, ^c^: p<0.05, when compared with Group 1. *: As Group 3 only included 7 rats there is one median value, the other groups comprised 8 rats and had 2 median values.

When Group 4 is compared with Group 1, there was no statistical significance for bleeding (p=0.428) and alveoloseptal thickening (p=0.085), while there were clear positive effects in Group 1 for cellular injury (p=0.030), inflammation (p=0.000) and total score (p=0.007).

When Group 4 is compared with Group 2 and Group 3, positive effects were only observed in Groups 2 and 3 for inflammation (p=0.020, p=0.012, respectively), with no statistical significance for the other parameters (p>0.05).

While there was no statistical significance for hemorrhage between Group 1 and Group 2 (p=0.655), there were clear positive effects in Group 1 for cellular damage (p=0.002), alveoloseptal thickening (p=0.044), inflammation (p=0.002) and total score (p=0.017).

**Figure 2:**
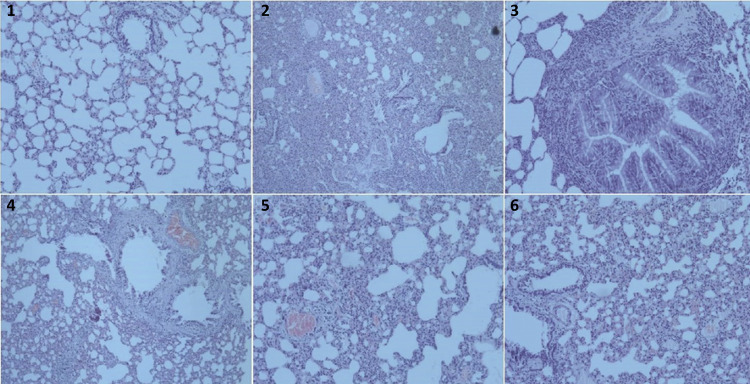
Some histopathologic section samples from the groups. 1. Group 5: Lung tissue at morphological limits (H&E, x100); 2. Group 4: Vascular congestion, severe intraalveolar hemorrhage and parenchymal inflammation (H&E, x100); 3. Group 4: Peribronchial inflammation (H&E, x200); 4. Group 1: Vascular congestion, moderate intraalveolar hemorrhage and parenchymal inflammation (H&E, x100); 5. Group 2: Vascular congestion and moderate parenchymal inflammation (H&E, x100); 6. Group 3: Vascular congestion and moderate parenchymal inflammation (H&E, x100).

When Group 1 is compared with Group 3, there was no statistical significance for bleeding (p=0.901), alveoloseptal thickening (p=0.081) and inflammation (p=0.054), but positive effects were pronounced in Group 1 for cellular injury (p=0.002) and total score (p=0.035). Histopathologic findings of the groups are demonstrated in Figure 2. Comparison of Groups 2 and 3 did not have any statistical significance (p>0.05).

## Discussion

Sepsis-linked acute lung injury is an important cause of morbidity and mortality in adults and children and significantly contributes to intensive care costs^17-20^. Many studies have created pioneering ideas for the use of ozone as a strong antimicrobial agent^21-24^. Ozone is a strong oxidizing agent and important disinfectant. According to literature data, exposure of bacteria, spores and viruses to ozone for only a few minutes causes inactivation^25-26^. Studies by Ricevudi *et al*.^27^ proposed oxygen-ozone treatment as a new immunotherapeutic treatment method for the viral agent COVID-19 causing this pandemic and that patients with COVID-19 using this in combination with other treatment methods would benefit from the assisting and synergic effects of ozone therapy. Again, as stated by Ricevudi *et al*.^27^ the need for more studies is clear. The bactericidal effect of ozone is linked to attacking the biological material in microorganisms through the oxidation pathway. In fact, the antibacterial effect of ozone is said to be more effective than iodine and chlorine^23,28^. Previous experimental studies have shown that ozone therapy has experimental benefits on pathologic processes^16,23,29^. In many organs like pancreas, peritoneum, liver, mesenteric lymph nodes and cecum, ozone therapy has proven reducing effects on bacterial translocation^16^. In an experimental necrotizing pancreatitis model, ozone therapy was seen to be more effective to reduce oxidative stress levels, tissue injury and bacterial translocation rates compared to hyperbaric oxygen treatment^30^. Schulz *et al*.^31^ observed a decrease in polymicrobial peritonitis after they administered intraperitoneal ozone. Çakır *et al*.^32^ reported the systemic inflammatory response markers of TNF-alpha and IL-1B levels reduced after ozone treatment. An experimental sepsis study by Yamanel *et al*.^16^ compared hyperbaric oxygen treatment with ozone therapy and identified that both methods lowered oxidative stress indices, myeloperoxidase activity and serum proinflammatory cytokine levels. As a result, histopathologic injury reduced in the lung tissue of septic rats and ozone therapy showed to provide more benefits according to histopathologic injury scores and IL-1B levels. As a result of the experimental study, they proposed that ozone therapy may be a complementary medicine treatment method that could be applied together with antibiotherapy for sepsis.

Additionally, studies have shown that ozone causes clear air pollution, and irritation and injury when taken in with immune and inflammatory cells in the lungs. This event progresses with bronchial epithelium desquamation and alveolar septal injury results in emphysema and airway hyperresponsiveness^33,34^. Ozone-linked injury and inflammation is linked to the dose and frequency of ozone exposure^35^.

In our study, positive effects were observed in terms of inflammation in Group 2 and Group 3 with added ozone therapy (p=0.020, p=0.012, respectively). However, it is considered that these effects are due more to antibiotic effects rather than to ozone therapy. The source point for this thought is that the cellular injury, inflammation, alveoloseptal thickening and general histopathologic scores in Group 1, only administered antibiotics, were at statistically significantly low levels compared to Group 2 (p=0.002, p=0.002, p=0.044, p=0.0017, respectively). Similarly, the cellular injury and general histopathologic score were observed at statistically significantly low levels in Group 1, administered only with antibiotic, compared to Group 3, administered with high-dose ozone (p=0.002, p=0.035, respectively).

When the results of our study are investigated, it was identified that ozone increased the negative histopathologic effects induced in the lung by inflammation. However, as the dose of ozone increased, the fall in the number of parameters with statistical significance confirms that positive effects may occur if the dose of ozone used was increased. Additionally, a limitation of our study is that there was no group administered with ozone therapy alone after sepsis, so the histopathologic effects of ozone on lung tissue in sepsis could not be assessed.

## Conclusion

Data obtained as a result of our study lead to the consideration that ozone therapy administered in addition to antibiotherapy may cause negative effects on lung tissue damaged due to sepsis.
